# Den Patienten wirklich verstehen lernen: Real-world-Evidenz aus der „patient journey“

**DOI:** 10.1007/s11553-022-00984-8

**Published:** 2022-10-14

**Authors:** Petya Zyumbileva, Maria Uebe, Stefanie Rudolph, Christof von Kalle

**Affiliations:** 1 Clinical Study Center (CSC) von Charité und BIH, Charité – Universitätsmedizin Berlin, corporate member of Freie Universität Berlin and Humboldt Universität zu Berlin, Charitéplatz 1, 10117 Berlin, Deutschland; 2 BIH Chair für Klinisch-Translationale Wissenschaften, Clinical Study Center (CSC) von Charité und BIH, Berlin Institute of Health at Charité – Universitätsmedizin Berlin, Charitéplatz 1, 10117 Berlin, Deutschland

**Keywords:** Patientenzentrierte Lösungen, Patientenberichtete Ereignisse, Standardisierung, Datensprache, Datenaustauschbarkeit, Patient journey, Patient-reported outcomes, Data standard, Common language, Data exchange

## Abstract

**Hintergrund:**

Die COVID-19-Pandemie („coronavirus disease 2019“) hat die Bedeutung von Real World Data (RWD) im klinischen Alltag unterstrichen und die fatalen Folgen von längst existierenden Problemen wie Lücken in der Primärdatenerfassung, Hürden bei der Auswertung von Patientendaten sowie erschwertem Patientendatenaustausch zwischen verschiedenen Einrichtungen nochmal deutlich gemacht. Darüber hinaus haben Entwicklungen weg von einem paternalistischen hin zu einem partnerschaftlichen Modell der Arzt-Patienten-Beziehung sowie die zunehmende Digitalisierung unser Verständnis von Gesundheitsversorgung geprägt, das Thema der Patientenautonomie und Selbstwirksamkeit in den Vordergrund gebracht und den Bedarf an innovativen, patientenzentrierten Lösungsansätzen verdeutlicht.

**Methoden:**

Wir nutzen die „patient journey“ als theoretisches Konstrukt, entlang dessen wir die Sammlung von verschiedenen Typen von RWD, ihre Bedeutung und Umgang damit beschreiben.

**Schlussfolgerung:**

Die Abbildung der „patient journey“ in Verbindung mit der Nutzung eines einheitlichen Datenstandards kann zur Erfassung von Primärdaten im Gesundheitswesen führen, die von allen medizinischen Behandlungseinrichtungen genutzt werden können. Dies wird den Austausch von Daten zwischen Einrichtungen erleichtern. Darüber hinaus könnte die fortlaufende Auswertung von patientenberichteten Ereignissen als Standard in der klinischen Routine die Patientenautonomie stärken und die Behandlung optimieren. Zusammenfassend lässt sich sagen, dass der Behandlungserfolg, das Gesamtüberleben und das Wohlbefinden der Patienten durch die Schaffung einer gemeinsamen Datensprache und eines ganzheitlichen, menschenzentrierten Ansatzes verbessert werden können.

## Abstract (en)

**Background:**

The coronavirus disease 2019 (COVID-19) pandemic has underscored the importance of real world data in everyday clinical practice and has highlighted some long-standing problems of our healthcare system such as gaps in primary data collection, hurdles in the evaluation of patient data, and complexity regarding the data exchange between different institutions. In addition, changes in physician–patient relationships such as transitions from a paternalistic to a partnership-based relationship model as well as increasing digitalization have shaped our modern understanding of healthcare, emphasizing the issue of patient autonomy and self-efficacy and highlighting the need for innovative, patient-centered approaches.

**Methods:**

Using the patient journey as a theoretical construct, we describe the collection of different types of real world data, their meaning and handling.

**Conclusion:**

Mapping the patient journey process combined with a widely used data standard can lead to the acquisition of primary data in the healthcare sector which can be used by all medical treatment institutions. This will lead to an exchange of valuable data between institutions and circuit the current problem of proprietary formats. Furthermore, the evaluation of patient-reported outcomes as a standard in the clinical routine could enhance patients’ autonomy and optimize treatment. Thus, the overall treatment effectiveness and survival of patients can be improved by creating a common data language and using a holistic, human-centered care approach through integrating perspectives of patients and their loved ones.

Die COVID-19-Pandemie („coronavirus disease 2019“) hat uns gezeigt, wie essentiell der Zugang zu Patientendaten und deren unkomplizierter Nutzung für die Forschung ist. Die bürokratischen Hürden bei der Erhebung und Auswertung von Real World Data (RWD) wurden bereits zum Ausbruch der Pandemie deutlich: Jede Beobachtungsstudie, die minimalinvasiv Virus- oder Impftiter erhebt, wurde zu einer Zulassungsstudie erhoben, die einen hohen Aufwand an Verwaltung hat. Solche Studien konnten dann in Deutschland nicht oder nur verspätet durchgeführt werden und man war auf die Daten aus anderen Ländern angewiesen. Dabei werden Daten bei jeder Station der „patient journey“ generiert und sind die Essenz einer personalisierten Versorgung.

Life is what happens to us while we are making other plans (Allen Saunders, Publishers Syndicate).

## Die Patient Journey im Überblick

**Abb. 1:**
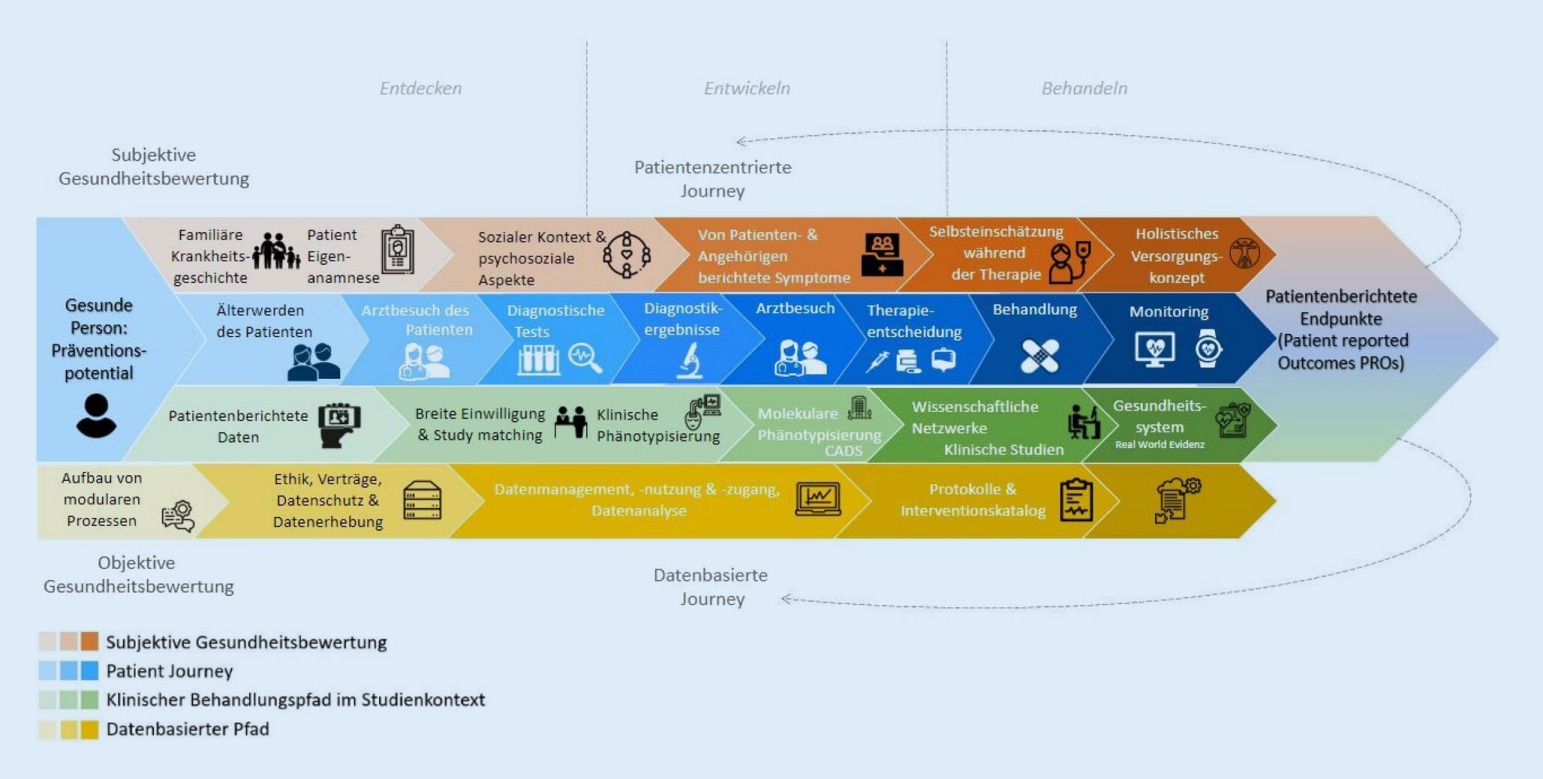
„Patient-journey“-Konzept von Real-world-Evidenz. (Diese Grafik wurde unter Verwendung von Symbolen von Flaticon.com erstellt.)

Die zunehmende Digitalisierung unserer Gesellschaft hat weitreichende Auswirkungen im Gesundheitswesen, insbesondere auf die Kommunikation zwischen Ärzten^1^, Patienten und ihren Angehörigen. Durch die stetig wachsende Informiertheit der Patienten über ihre Erkrankung(en) entwickelt sich die zuvor passive Rolle der Patienten zu einer aktiven, mitentscheidenden und weiterbildenden Rolle. Das zuvor strikte Rollenkonzept Arzt-Patient kann sich so in naher Zukunft zu einem eher partnerschaftlichen Modell weiterentwickeln. Allerdings sind der tatsächliche Zugang des Patienten zu seinen eigenen Daten und die damit einhergehende Stärkung seiner Souveränität im Umgang mit diesen noch im Anfangsstadium. Abb. 1 illustriert den Zusammenhang zwischen der Patient Journey, der subjektiven Wahrnehmung des Patienten und seinem Umfeld, sowie den Datenfluss, der mit der Behandlung eines Patienten einhergeht.

Die „patient journey“ stellt die zeitliche Abfolge der Phasen dar, die der Patient während seiner Versorgung durchläuft [3]. Jeder Punkt der Patientenreise bietet Daten über Gesundheitsergebnisse und Patientenerfahrungen, die als Feedback genutzt werden sollten, um die Prozesse im Gesundheitswesen im Sinne von Anpassung, Lernen und Verbesserung neu zu gestalten.

In den nachfolgenden Abschnitten werden die z. T. (aber nicht zwingend) aufeinander aufbauenden Stationen der „patient journey“ näher erläutert. Präventionsmaßnahmen wie auch Maßnahmen der „disease interception“ können als Möglichkeiten der Vermeidung von Erkrankungen dienen bzw. des frühzeitigen Eingriffs in eine Erkrankung, um deren Fortschreiten zu verhindern.

Die anschließenden Ausführungen zu Diagnose, Therapien und dem Follow-up beschreiben die meist aufeinander aufbauenden Stadien einer „klassischen patient journey“ vom Eintritt in den Behandlungszyklus bis zum Abschluss bzw. Wiedereintritt.

Die Abb. 1 soll verdeutlichen, dass neben der subjektiven Wahrnehmung des Patienten eine leitliniengetreue Behandlung in Deutschland im Vordergrund steht. Im gesamten Prozess werden Daten des Patienten aufgenommen und verarbeitet, ausgewertet und für weitere Behandlungen eingesetzt. Jedoch decken die „harten Fakten“ wie Auswertungen von radiologischen Bildern und pathologischen Befunden nicht unbedingt die subjektiven Wahrnehmungen des Patienten über den eigenen Zustand ab, wie Abgeschlagenheit, Müdigkeit und allgemeines (Un)wohlsein. Daher müssen alle Wege – die der Daten, der subjektiven Eindrücke des Patienten, aber auch die leitliniengetreue Ausführung von Therapien ineinandergreifen. Durch dieses Zusammenspiel und eine gemeinsame Auswertung können wiederum Handlungsempfehlungen für weitere Therapien und Diagnostik entwickelt werden.

### Prävention als Erststation der „patient journey“

Die „patient journey“ beginnt nicht erst, wenn der Patient erste Symptome einer Erkrankung aufweist, sondern bereits vor dem Auftreten einer Erkrankung; bei der Primärprävention. Diese umfasst Maßnahmen, die auf die Identifikation und Vermeidung von Gesundheitsrisiken abzielen und die Entstehung von Krankheiten verhindern [15]. Die sekundäre Prävention zielt auf die Früherkennung ab, sodass Erkrankungen zu einem möglichst frühen Zeitpunkt erkannt werden, um so eine frühzeitige Therapie einleiten zu können. Die letzte Stufe der Prävention umfasst alle tertiären Maßnahmen, um Krankheitsfolgen zu mindern, Rückfälle zu vermeiden und die Verschlechterung des Gesundheitszustands zu verhindern. Diese Maßnahmen können z. T. mit Rehabilitationsmaßnahmen verglichen werden, wobei diese noch spezifischer darauf abzielen, die Funktionsfähigkeit zu optimieren und Behinderungen bei Menschen mit gesundheitlichen Einschränkungen in Wechselwirkung mit ihrer Umwelt zu verringern [34]. Um einen ganzheitlichen Ansatz einer „patient journey“ zu verfolgen, sollten auch diese an das Ende einer „patient journey“ gekoppelt sein.

### „Disease interception“

Ein noch recht neues Konzept stellt die sog. „disease interception“ dar. Gerade im Bereich der Onkologie oder des Diabetes mellitus kann diese wertvolle Zeit gewinnen, da hier der Fokus auf personalisierten Interventionen liegt, die den Ausbruch oder das Fortschreiten einer Erkrankung verhindern sollen [2]. Das Konzept stützt sich insbesondere auf die Erkennung von genetischen Faktoren und Biomarkern zur zielgerichteten Therapie, zugeschnitten auf jeden einzelnen Patienten und seine Anforderungen. Genom- und Proteomanalysen können genetische Prädispositionen ermitteln und so Hochrisikopatienten rechtzeitig helfen [2].

### Symptome und Diagnose

Jede „patient journey“ beginnt nicht zwingenderweise direkt mit der Diagnose der Erkrankung, sondern kann über mehrere Etappen von diffusen Symptomen, uneindeutigen Beschwerden bis hin zu unklaren Befunden und versuchter Diagnosefindung bei unterschiedlichen Ärzten reichen. Im Laufe des Diagnosestellungsprozesses können umfangreiche Tests und Analyseverfahren von Nöten sein, welche verschiedenste Verfahren wie Laboruntersuchungen, Histopathologie, Bildgebung und molekulare Diagnostik umfassen. Diese komplexe Bestandsaufnahme und die resultierende Diagnose geben oftmals den Weg für eine anschließende therapeutische Behandlung frei.

### Therapie und Behandlung

Eine Therapie kann unterschiedliche Handlungsstränge aufweisen, welche teils zeitlich hintereinander, aber auch parallel zueinander erfolgen können. Eine kontinuierliche Analyse des Outputs des Therapie- oder Misserfolgs ist hierbei notwendig, um das therapeutische Ansprechen auszuwerten, mögliche Resistenzentwicklungen rechtzeitig festzustellen und gegebenenfalls auch neue angepasste Therapieempfehlungen herzuleiten. Darüber hinaus können Erkenntnisse aus der Routineversorgung die Sicherheit beim Umgang mit Nebenwirkungen von medikamentösen Therapien erhöhen und die Entwicklung von Therapieleitlinien unterstützen [32]. Die routinierte Erfassung und Auswertung von patientenberichteten Ereignissen während der akuten Behandlungsphase kann zu einer Früherkennung von Wechsel- und Nebenwirkungen und erfolgreichem Symptommanagement beitragen [17].

### Follow-up

Nach erfolgter Behandlung folgt oftmals ein Follow-up, um die weitere Entwicklung der Erkrankung des Patienten in regelmäßigen Abständen zu kontrollieren und entsprechend zu überwachen. In der Onkologie spielt die Nachsorge eine wichtige Rolle bei der rechtzeitigen Erkennung von Tumorrückfällen oder Metastasen. Leider ist es in vielen Fällen so, dass es gerade in der Nachsorge zu erheblichen Informationsverlusten kommt. Gründe hierfür reichen von nicht eingehaltenen Nachsorgeuntersuchungen aufgrund von fehlenden Beschwerden oder Angst vor möglichen (negativen) Ergebnissen seitens des Patienten über den Wechsel des Behandlungsortes bis hin zur Nicht-Auffindbarkeit bzw. den Tod des Patienten [13]. Andere Gründe können aber auch beim Behandler liegen, wie das Festhalten an starren Nachsorgeleitlinien ohne auf die individuellen Bedürfnisse des Patienten einzugehen. Zudem ist die Übergabe eines gut durchdachten Nachsorgeplans mit Kontakten zu weiterbehandelnden Fachbereichen und zusätzlichen Hilfsangeboten, die über die rein ursachenbezogene Therapie hinausgehen, wichtig. Hierunter fallen beispielsweise eine psychologische Betreuung, Ernährungsberatung oder die Beantragung einer Kur oder Rehabilitationsmaßnahme. Diese Probleme benötigen individualisierte Lösungen, die z. T. von den standardisierten Leitlinienkonzepten abweichen können.

## Patientenzentrierte Lösungsansätze im Gesamtablauf – auf das Individuum angepasste Medizin

Die Kopplung der einzelnen Ereignisse der „patient journey“ zu bestimmten Zeitpunkten ist notwendig, um zu dokumentieren, welche Maßnahmen die Erkrankung auf welche Weise beeinflusst haben. Diese longitudinale Datenerfassung ermöglicht es zeit- und eventgebundene Geschehnisse miteinander ins Verhältnis zu setzen und dient als Grundlage für die Entwicklung von patientenzentrierten Lösungsansätzen.

### Integration von „patient reported data and outcomes“ (PRO)

Um eine individualisierte Medizin ganzheitlich zu ermöglichen, muss eine systematische Berücksichtigung der Patientenperspektive bereits vor Beginn der Behandlung in Form von Eigenanamnese und Erfassung der familiären Krankheitsdisposition erfolgen. Im Rahmen der Erhebung der subjektiven Wahrnehmung des Krankheitsverlaufs seitens der Patienten und seiner Angehörigen sowie bei der systematischen Erfassung von patientenberichteten Ereignissen mit Hilfe von standardisierten Messinstrumenten („patient reported outcome measures“, PROM) wird RWD (subjektiv und objektiv) generiert. Unter RWD werden Daten verstanden, die in einer nicht-interventionellen bzw. nicht-kontrollierten Umgebung routinemäßig in Form von elektronischen Gesundheitsakten (EHR), Patientenregistern, Wearables, genomischen Datensätzen, Registern für medizinische Leistungen erhoben werden (d. h. Daten, die ohne Beeinflussung der Behandlungszuweisung und/oder der Patientenüberwachung/Nachbeobachtung und/oder der Auswahl der Studienpopulation erhoben werden; [21, 22]).

Diese Form der Einbindung in die Behandlung befähigt die Patienten, sich kontinuierlich und aktiv an ihrem Gesundheitsmanagement zu beteiligen und kann zugleich die therapeutische Adhärenz erhöhen [5, 16]. Dabei können PRO als Lösungsansatz über die Behandlungsphase hinaus herangezogen werden. Die digitale Erfassung von PRO im Follow-up kann einen Überlebensvorteil für Krebspatienten bringen, da diese die frühere Erkennung von Rezidiven ermöglicht [12]. PRO wie Lebensqualität, Schmerz und Fatigue können den zu erwartenden klinischen Verlauf vorhersagen und als unabhängige prognostische Faktoren für das krankheitsfreie Überleben bei manchen Krebsarten fungieren [6, 10, 20, 29].

Die personalisierte Medizin soll die vielfältigen Bedürfnisse von Krebspatienten und ihren Familien abdecken. Im Rahmen eines gesamtheitlichen Behandlungskonzepts sollte daher auch die duale Perspektive von Patienten und Angehörigen als eine dyadische Betrachtung herangezogen werden. Die bei chronischen Erkrankungen häufig zeitlich zunehmende psychische Belastung der pflegenden Angehörigen und die oftmals damit verbundene Verschlechterung ihres körperlichen Allgemeinzustands haben unmittelbare Auswirkungen auf das körperliche und mentale Wohlbefinden des Patienten [19]. Die Berücksichtigung der häuslichen Pflegesituation kann daher eine individualisierte Herangehensweise ermöglichen. Emotionale, soziale und finanzielle Auswirkungen (wie z. B. finanzielle Schwierigkeit [31], Isolation [23], Demoralisierung [14]) stellen relevante Bereiche dar, die bisher unzureichend adressiert werden und die ebenfalls bedeutende Aspekte innerhalb der „patient journey“ darstellen.

### Stärkung der Selbstwirksamkeit durch patientenzentriertes Gesundheitsdatenmanagement

Um die Erfassung und Auswertung von patientenzentrierten Ereignissen mit den Behandlungsdaten zu verknüpfen, ist die Errichtung von individuellen Datenräumen, die von den Patienten selbst verwaltet und mit bestimmten Gesundheitsdienstleistern ihrer Wahl geteilt werden, notwendig. So soll ein intersektoraler Gesundheitsdatenaustausch entstehen, der nicht nur die Stärkung der Patientensouveränität und den Aufbau eines digitalen Patientenserviceökosystems ermöglicht, sondern auch nachhaltige Lösungen für den Datenumgang schafft. Die Einführung einer sog. breiten Einwilligung („broad consent“) ermöglicht es, alle Gesundheitsdaten, die eine entscheidende Rolle in der Behandlungssituation spielen, systematisch, fortlaufend und lückenlos zu sammeln und auszuwerten. So bleibt das Verständnis von „personalisierter Medizin“ nicht nur ein Teil der Grundlagenforschung, sondern wird im klinischen Alltag als eine moderne Versorgungsform gelebt.

## Aktueller Stand der proprietären Primärdatenerfassung in der Versorgung

Zu jedem Zeitpunkt der oben beschriebenen Stationen der „patient journey“ wird eine Vielzahl von Daten erhoben, die im proprietären^2^, meist nicht austauschbaren Format existieren. Dies sorgt dafür, dass im Studienkontext bereits erfasste Daten für andere Interessierte aus der Forschung nicht zur Verfügung stehen. Aufgrund der Nutzung unterschiedlicher Datenformate können dabei essentielle Daten bei der Weitergabe verloren gehen. Die Überführung von Papierakten in ein digitales Format kann nur unter großem Zeitaufwand mit potenziell hoher Fehlerquote vollzogen werden. Aus diesem Grund wird eine sektorenübergreifende Digitalisierung des Gesundheitssystems mit semantischen und syntaktischen Standards dringend benötigt. Erfolgreiche Beispiele für solche Standards sind die sog. „fast healthcare interoperability resources“ (FHIR), die den Datenaustausch zwischen verschiedenen Softwaresystemen erleichtern und interoperable Lösungen ermöglichen [26].

## Datenaustauschbarkeit durch eine gemeinsame Datensprache

Um ein gemeinsames Verständnis und eine Nutzung von bereits vorhandenen und zukünftigen Daten zu schaffen, benötigt das Gesundheitssystem eine gemeinsame Datensprache. Sie muss technische Wirksamkeit aufweisen und somit mit allen benutzten Datenformaten kompatibel sein und gleichzeitig alle individuellen, inhaltlichen Aspekte verschiedenster Akteure innerhalb des Gesundheitssektors abbilden können. International anerkannte Kodesysteme wie SNOMED CT („systematized nomenclature of medicine clinical terms“) oder LOINC („logical observation identifiers, names, and codes“) bieten hierbei gute Lösungen, die durch iterative und ständige Erweiterung den neuesten Stand der Wissenschaft abbilden [8].

## Instrumente der Real-world-Evidenz

Die RWD, die routinemäßig erhoben werden, können aggregiert, verknüpft und verarbeitet werden, um wichtige Schlussfolgerungen in Form von Real-world-Evidenz (RWE) zu ziehen [22]. Die Nutzung von RWE im Gesundheitssystem ist essentiell, um Erfolgsquoten von Therapien im klinischen Alltag messen zu können. Im Fall von streng kontrollierten Studien, die in einem vordefinierten Rahmen ablaufen ist dies nicht immer möglich. Als Beispiel kann hier die aktuelle Impfstoffentwicklung während der COVID-19-Pandemie herangezogen werden. Obwohl randomisierte klinische Studien als „Goldstandard“ gelten, weisen sie gewisse Einschränkungen bezüglich der Stichprobengröße und Subgruppenanalyse auf. Die restriktiven Einschlusskriterien, die eine stark kontrollierte Umgebung erzeugen, sind beispielsweise bei einer Massenimpfung möglicherweise nicht reproduzierbar. So wurden bei Phase-III-Studien mit dem BNT162b2 mRNA-Impfstoff gegen COVID-19 21.720 Personen eingeschlossen [27], die nach dem Zufallsprinzip der geimpften Gruppe zugewiesen wurden, was eine Beurteilung der Wirksamkeit des Impfstoffs nur in einer kleinen Anzahl von Teilpopulationen erlaubt [11]. Dabei wurden nur Patienten mit chronischen Krankheiten eingeschlossen, wenn ihr Zustand als stabil eingestuft wurde [7]. Um nicht nur einzelne Gruppen, sondern die Gesamtbevölkerung zu erreichen, den Impfschutz auszuwerten und vergleichen zu können, wurde in Israel eine große, nationale Beobachtungsstudie aufgesetzt. Diese berücksichtigte unter anderem auch Aspekte in realen Settings, ob in einem aufgestockten Impfprogramm Faktoren wie Aufrechterhaltung der Kühlkette und die Impfstoffbereitstellung sowie suboptimale Einhaltung von Impfplänen und Logistik der Impfstoffhandhabung die Impfstoffwirksamkeit beeinflussen würden. So wurden jeden Tag im Zeitraum vom 20. Dezember 2020 bis zum 01. Februar 2021 alle neu geimpften Personen im Verhältnis 1:1 mit ungeimpften Kontrollpersonen verglichen [11]. Dabei konnten neu geimpfte Personen in die Studie aufgenommen werden, auch wenn sie zuvor als Kontrollgruppe ausgewählt worden waren [11]. Solche Studiendesigns, die sowohl den Charakter von Anwendungsbeobachtung haben, indem sie Daten aus der Versorgung nutzen, als auch Elemente von randomisierten Studien (Fall-Kontroll-Gruppen) beinhalten, stellen eine effektive Methode zur Generierung von RWE dar und sind ein nicht mehr wegzudenkendes Instrument bei der Bewältigung von künftigen Pandemien.

Nicht nur in der Pandemiebekämpfung, sondern vor allem im Umgang mit seltenen Erkrankungen ist die Nutzung von RWD unabdingbar. Aufgrund der verhältnismäßig kleinen Patientenpopulationen gestaltet sich hier die Durchführung von klassischen randomisierten Phase-II- und Phase-III-Studien schwierig. 90 % der Patienten mit seltenen Erkrankungen erhalten mindestens ein Medikament, das nicht für ihre Indikation zugelassen ist, das sog. „off-label use“ [25]. Off-label-Therapien spielen auch in der Pädiatrie eine wichtige Rolle: Drei Viertel der auf dem Markt zugelassenen verschreibungspflichtigen Arzneimittel haben keine Indikation für Kinder, sodass ihre Verwendung dem Ermessen des Pädiaters überlassen bleibt [4]. Die Anwendung von Beobachtungsstudien mit registerartigen Funktionen, in denen alle Daten über Patienten mit seltenen Erkrankungen vollständig erfasst und aktualisiert werden, kann hierbei sehr hilfreich sein. So lässt sich aktuelles Wissen über Symptome, Krankheitsverläufe und Wirksamkeit von Therapien fortlaufend generieren, die Erkrankungen und deren Pathogenese besser verstehen, neue Targets identifizieren und anekdotische Evidenz ableiten.

### Auswertungsmethoden auf Basis von RWD

Die innovative Auswertung von datenintensiven Abläufen ist insbesondere bei seltenen Erkrankungen, die häufig eine genetische Ursache haben, notwendig, um Erkenntnisse über Behandlungsmöglichkeiten zu gewinnen [33]. Zur Generierung von RWE können komplexe Verfahren der Genomanalyse eingesetzt werden, die die Verkürzung des Diagnoseweges erlauben und Anhaltspunkte für die Therapieplanung liefern. Hierbei können Sequenzierungstechniken wie das „next generation sequencing“ (NGS) zur Abklärung unklarer Diagnosen beitragen [24]. Digitale Methoden, die auf künstlicher Intelligenz (KI) basieren, können das Prozessieren von großen Datenvolumen wie beispielsweise in der Radiologie unterstützen. Eine effizientere Befundung bei steigender Anzahl von Untersuchungen und Bilddatenmengen kann durch KI-Verfahren bewältigt werden und dabei die Qualität sichern und zu besserer Diagnostik führen [9]. Die Verwendung von KI kann das Treffen von gesundheitspolitischen Maßnahmen auch bei pandemischen Lagen unterstützen. So zeigte die Anwendung eines mathematischen Modells in der frühen Phase des COVID-19-Ausbruchs in Deutschland, welches epidemiologische Modellierung mit spezialisierten neuronalen Netzen kombiniert, dass zuverlässige probabilistische Schätzungen für wichtige Merkmale wie z. B. Generationszeit, Anteil der unentdeckten Infektionen, die Übertragungswahrscheinlichkeit generiert werden können [28]. Weitere internationale Studien belegen die Genauigkeit der Vorhersagekraft von ähnlichen KI-gestützten Modellen und unterstreichen die unterstützende Rolle von solchen Simulationsverfahren beim Treffen von Präventivmaßnahmen und wissenschaftsbasierten politischen Entscheidungen [18, 30].

Die Nutzung von RWE kann nicht zuletzt die Qualität der medizinischen Versorgung sicherstellen. Ihr Einsatz kann die Sicherheit und Nebenwirkungen von Medikamenten überwachen und zur rechtzeitigen Erkennung von Krankheitsmustern beitragen [27]. Folglich kann die Erweiterung von Therapieindikationen sowie die Entwicklung von neuen Therapierichtlinien unterstützt und eine individualisierte und patientenzentrierte Versorgung ermöglicht werden.

## Schlussbetrachtung

Um Anschluss an die moderne Medizin halten zu können, muss die Auswertung von RWD über die gesamte Patient Journey hinweg erlaubt und vereinfacht werden, so kann auf lange Sicht ein lernendes, patientenzentriertes Gesundheitssystem entstehen. Die Errichtung individueller, digitaler Datenräume, auf die der Patient nicht nur Zugriff hat, sondern diese selbstständig verwaltet, dabei bestimmt, wer auf welche Informationen zugreifen kann und den Überblick über seine eigenen Daten behält, wird die Patientensouveränität stärken und zu einem digitalen Patientenservice-Ökosystem beitragen. Das in Deutschland herrschende Verständnis von Datenschutz als Nicht-Prozessieren von Daten^3^ kann erhebliche negative Konsequenzen haben und dazu führen, dass Patienten zu Schaden kommen. Die bisherigen ethischen Überlegungen des aktuellen Datenschutzmodells müssen also neu überdacht und patientenorientiert gestaltet werden. Im Rahmen eines translationalen Netzwerkmodells von verknüpften, leistungsstarken akademischen und ggf. auch kommerziellen Forschungseinrichtungen, welches zusammen mit Kliniken, medizinischen Versorgungszentren, Niedergelassenen und sonstigen Gesundheitseinrichtungen kooperiert, kann die Auswertung der in der Routineversorgung erhobenen Daten erfolgen. Darüber hinaus können neu gewonnene Forschungsergebnisse, die Ausgangspunkte für zukunftweisende, maßgeschneiderte Therapien und neue patientenzentrierte, individualisierte Behandlungsansätze bieten, die auf Real-world-Evidenz basieren, in die Versorgung zurückgespeist werden und Innovation an das Krankenbett bringen, wovon alle Patienten profitieren.

## Fazit für die Praxis

- Die Einführung einer so genannten breiten Einwilligung kann die systematische und lückenlose Erfassung und Auswertung der persönlichen Gesundheitsdaten für Versorgung und Forschung ermöglichen. Auch hier bleibt der Patient derjenige, der bestimmt, ob seine Daten für medizinische Forschungszwecke genutzt werden dürfen.
- Innovative (klinische), adaptive Studiendesigns, die gleichzeitig Elemente von Anwendungsbeobachtungen und randomisierten kontrollierten Studien (z. B. externe Kontrollarme) einbeziehen und die Analyse von Real World Data erlauben, sollen als Vorbild verwendet werden.
- Eine gemeinsame Datensprache und interoperable Formate sollen zum intersektoralen Austausch von Patientendaten und Schaffung von Real-world-Evidenz herangezogen werden.
- Es soll eine ganzheitliche Betrachtung der Patient Journey etabliert werden, die die subjektive Wahrnehmung der Patienten und Angehörigen integriert und neben krankheitsspezifischen auch psychosoziale Aspekte berücksichtigt.

## Footnotes

1 Aus Gründen der besseren Lesbarkeit wird im Folgenden auf die gleichzeitige Verwendung weiblicher und männlicher Sprachformen verzichtet und das generische Maskulinum verwendet. Sämtliche Personenbezeichnungen gelten gleichermaßen für beide Geschlechter.

2 „Man bezeichnet im IT-Bereich traditionell Dateiformate, Protokolle usw. aber auch Hardware als proprietär, die nicht allgemein anerkannten Standards entsprechen“ [1].

3 Unter nicht-prozessierten Daten werden Rohdaten, also unstrukturierte Daten verstanden, die beispielsweise innerhalb der Versorgung gesammelt werden, aber nicht weiter aufbereitet oder verfügbar gemacht werden, um sie in anderen Bereichen wie der Forschung weiterzuverwenden.
